# Clinical Reasoning: Defining It, Teaching It, Assessing It, Studying It

**DOI:** 10.5811/westjem.2016.11.33191

**Published:** 2016-12-05

**Authors:** Larry D. Gruppen

**Affiliations:** University of Michigan Medical School, Department of Learning Health Sciences, Ann Arbor, Michigan

Clinical reasoning is a perennial focus of medical education, performance assessment, and study. It might be argued to be the defining characteristic of the profession. It is, however, a very complex and multi-faceted phenomenon that can create considerable confusion and cross-communication. Its importance makes it worthwhile to consider some of those complexities.

## Defining it

Like the fable of the blind men and the elephant, each of whom, feeling a different part of the elephant, described it in very different ways, clinical reasoning is a vast, complex construct that is described and used in different ways by different people. There is no generally accepted definition of clinical reasoning and, indeed, many articles about clinical reasoning never define it explicitly; it is often assumed as a universally understood construct. For the present commentary, we can describe the clinical reasoning process as including the physician’s integration of her own (biomedical and clinical) knowledge with initial patient information to form a case representation of the problem. The physician uses this problem representation to guide the acquisition of additional information and then, on the basis of this information, revises the problem representation. She repeats the information gathering – representation revision cycle until she reaches a threshold of confidence in that representation to support a final diagnosis and/or management actions.^1^ This very broad description subsumes numerous additional phenomena and questions: how is knowledge organized and accessed, how does expertise manifest itself in clinical reasoning, how are alternative representations evaluated, and so forth.

It is readily apparent to anyone reading the literature that “clinical reasoning” is used for a considerable variety of activities. Indeed, a skeptic may well ask “what is NOT clinical reasoning?” If the term comes to encompass any physician thinking about clinical problems, the concept becomes so expansive as to risk becoming useless as a guide to teaching, assessment and study. It is unlikely that we will achieve a clean taxonomy of clinical reasoning activities anytime soon, so in the meantime, it is important for anyone trying to teach, assess, or study clinical reasoning to recognize the complexity of the terms and be explicit about their operational definition.

In spite of this conceptual sprawl, there are still significant aspects of clinical reasoning that are largely ignored in the literature. Because it is often defined in terms of cognition, such things as context, affect, and institutional factors have rarely been examined for relevance to clinical reasoning. There is, however, a growing awareness of the importance of context and the larger system in which clinical reasoning takes place.^2^ Thinking about clinical reasoning as if it were isolated in the physician’s head is no longer viable.

Another aspect of clinical reasoning that has suffered significant neglect is management–attention is primarily devoted to diagnostic reasoning, not therapeutic reasoning. The preoccupation with diagnostic tasks is understandable. There is the prospect of a “correct” diagnosis and the attraction of being able to classify reasoning as successful or unsuccessful is undeniable. If one can be “scored” as right or wrong, all the reasoning steps that led up to that answer can be examined in the same right-wrong light. In contrast, therapy is much more difficult to classify as “right” and “wrong.” It depends on many variables that can be combined in numerous ways and it is often proven right or wrong only in hindsight. Individual physicians can make plausible arguments for very different management alternatives. It is much more a “matter of opinion” or judgment than a universally correct solution.

## Teaching it

Considerable effort goes into teaching clinical reasoning. Sometimes, this is the focus of specific courses, but it is a key goal of almost any course, clerkship or clinical rotation. Numerous innovations have been developed for teaching various aspects of clinical reasoning using carefully designed and selected cases, mnemonics for gathering information, identification of critical information to discriminate among diagnostic alternatives, appropriate methods for judging and managing uncertainty, de-biasing methods, and the like. These interventions are often designed to address common problems that learners demonstrate in clinical reasoning: inadequate knowledge, faulty data gathering, faulty data processing, or faulty metacognition.^3^

A risk in all of these efforts is that we come to believe we are teaching “clinical reasoning” as a generalizable skill that can be applied to any clinical problem. Unfortunately, this fond hope has little empirical support. From the earliest studies of medical problem solving^4^,^5^ to the present, the most reproducible result is that clinical reasoning performance is highly content (and context) specific. Solving a clinical problem in one discipline holds little predictive value for how one will do with a problem in another area. Even in problems with the same diagnosis, there is little consistency in performance. It is apparent that “reasoning skills” or “critical thinking” do not go far in helping develop clinical reasoning. Instead of general processes, it is knowledge that is key to performance. Indeed, most educational interventions that focus on clinical reasoning are also (perhaps implicitly) conveying knowledge in critical areas of medicine and it is this knowledge acquisition that fosters better performance.

At the extreme, this can be seen in the development of pattern recognition, in which knowledge of common patterns and relationships among information lead to recognition of disease possibilities WITHOUT conscious reasoning. Indeed, some do not consider “mere” pattern recognition as a manifestation of clinical reasoning simply because it bypasses the conscious, effortful thought processes and relies on automated cognitive processes.^6^ Clinical reasoning extends well into non-conscious as well as conscious processes.

## Assessing it

Numerous methods have been developed to assess clinical reasoning – or some part of it. A few examples are provided in the table. Each method addresses a component of the larger clinical reasoning process, often in the form of focusing on a particular sub-task, such as information gathering, adjusting diagnostic hypotheses for new information, using basic science knowledge to reason through an electrolyte problem, or prioritizing diagnostic alternatives. Each assessment method makes assumptions about the underlying construct (clinical reasoning) that must be considered before making general conclusions about an examinee’s competence.

Like teaching clinical reasoning, assessing it confronts the vexing phenomenon of content specificity. Even more challenging is the growing recognition that, even within the same content domain, the context of the task influences performance. Context includes psychological variables, such as fatigue and stress or immediately preceding patient experiences, social variables, such as team relationships and support, and institutional/environmental factors, such as inpatient vs. outpatient setting.^7^

## Studying it

As might be predicted from the centrality of clinical reasoning, there is a substantial body of research associated with it. This research can be divided into two broad perspectives – a *descriptive* perspective that focuses on the actual cognitive activities and actions of physicians while engaged in clinical reasoning, and a *prescriptive* perspective that defines optimal, rational models for reasoning and investigates how and to what extent physicians deviate from these normative models.

The descriptive perspective has its roots in cognitive psychology and began as a special case of general problem-solving studies. It focuses on clinical reasoning as a domain in which the problems are complex and there is a clear role for expertise. The critical role of knowledge distinguishes medicine from many other domains of problem-solving research, such as games, mathematics or logic, in which a relatively small number of rules were adequate for correct solutions. Descriptive studies often highlight four research themes: knowledge organization, cognitive processes, problem structure, and expertise characteristics.

Knowledge organization is a lynchpin of research on cognition generally and this interest extends to medicine as well. Theories of knowledge organization posit a wide range of explanatory constructs (prototypes, schemas, scripts, mental models, networks, etc.) and address questions about knowledge acquisition, retrieval and transfer. Many of these cognitive theories have concentrated on the use of knowledge rather than its acquisition, but educational theories of how knowledge is best acquired are also common in medical education.

A great deal of the research on clinical reasoning addresses the various cognitive processes involved. For example, foundational processes such as perception turn out to be essential to expertise. Experts “see” the world differently from novices by virtue of sophisticated “pattern recognition” capabilities that effectively move some of their knowledge to the unconscious, rapid, and automated process of perception. Attention is another cognitive process in which clinical expertise has an advantage in focusing on relevant information and not getting distracted by irrelevancies. Information gathering and evaluation are other critical cognitive processes that drive many studies. Comprehending and building a cognitive representation of a clinical problem are more advanced cognitive processes that are also heavily influenced by underlying knowledge. There are other cognitive processes and numerous theories that inform and stimulate a wealth of research questions.

The prescriptive perspective on clinical reasoning has its roots in computer science, economics, and probability theory. These disciplines provide the normative models for dealing with uncertainty, modeling complex decision alternatives, and balancing competing values. In comparison to these normative models, people (including physicians) are often irrational, illogical, and badly flawed reasoners. They regularly violate many of these normative principles and make predictable errors (biases) because they use simple shortcuts (i.e, heuristics).

The flawed (from the prescriptive perspective) nature of clinical reasoning leads to two kinds of research. One is the investigation of the conditions under which physician reasoning is more or less problematic and understanding how these errors and biases emerge. Often, the objective is to improve reasoning through educational interventions (e.g., de-biasing techniques). The second is to improve reasoning through decision support tools or computer-based programs that relieve physicians of many of the components of reasoning that produce errors. Decision support tools and reasoning models may be diagnostic or therapeutic in focus and are promoted as ways to reduce the undesirable variability in physician decisions that arise from faulty and inconsistent reasoning.

In summary, clinical reasoning is something of a “god term,” which supersedes and dominates many subordinate terms and concepts.^8^ Its “power” leads to rather indiscriminate and unthinking use which, in turn, contributes to confusion and conflicting discussions of the nature and function of clinical reasoning. If nothing else, I hope this commentary contributes to recognizing that we need to be careful about what we mean when we talk about clinical reasoning. We need to be more precise in defining what aspect of clinical reasoning we are interested in. We also need to use theory to help frame our thinking about this complex construct. Arguments about which is the “right” theory are moot – there is no one right way to think about clinical reasoning, but all will benefit from complementary perspectives that each contribute a piece to the greater puzzle.

## Figures and Tables

**Table:** Methods of assessing clinical reasoning.

Assessment method	Description
Chart stimulated recall	Using the patient chart generated by the clinician, probe for recall of the reasoning process in connection with key elements of the chart.
Concept map	Graphic representation of knowledge constructs and relationships among them (organization). Used for both teaching and assessment.
Direct observation	Observation in a clinical reasoning task and judgment of performance against specified criteria.
Extended matching, multiple choice questions	Select best response from a restricted number of alternative answers. Most commonly used to asses knowledge, but amenable to more sophisticated tasks.
Patient management problems	A structured patient case that allows flexible selection of clinical information and the development of a dynamic diagnostic or management decision.
Post-encounter note	Written summary of patient case, relevant information, diagnosis, and treatment plan.
Script concordance tests	Assesses the impact of new information on a diagnostic hypothesis or the probability pursuing a specified action.
Simulation, standardized patients	Structured patient case with a trained actor that requires the learner to do a history and physical examination and generate a diagnostic solution.
Think aloud, oral exam	Verbalize one’s reasoning process as one works through a clinical case or specified problem – with or without prompts and probes from an examiner.
